# Head-to-Head Comparison of Single- Versus Dual-Chamber ICD Discriminators for Tachyarrhythmia Detection: A Single-Manufacturer, Remote Monitoring-Based Bicentric Study

**DOI:** 10.3390/jcm14165859

**Published:** 2025-08-19

**Authors:** Flora Diana Gausz, Daniel Fodor, Mirjam Turani, Marton Miklos, Attila Benak, Dora Kranyak, Attila Makai, Gabor Bencsik, Peter Bogyi, Robert Pap, Laszlo Saghy, Attila Nemes, Tamas Szili-Torok, Gabor Zoltan Duray, Mate Vamos

**Affiliations:** 1 Department of Internal Medicine, Cardiology Center, Division of Electrophysiology, University of Szeged, 6725 Szeged, Hungary; 2 Biotronik Hungary Ltd., 1124 Budapest, Hungary; 3 Department of Cardiology, Central Hospital of Northern Pest–Military Hospital, 1134 Budapest, Hungary

**Keywords:** ICD, implantable cardioverter defibrillator, tachycardia discrimination, morphology discrimination, remote monitoring

## Abstract

**Background**: Modern implantable cardioverter-defibrillators (ICDs) utilize single-chamber (SC) or dual-chamber (DC) discrimination algorithms to differentiate between tachyarrhythmias and minimize the risk of inappropriate therapies. While modern SC algorithms, especially those with morphology detection, are considered comparable to DC algorithms, the available data are limited. We aimed to compare the efficacy of SC and DC discrimination algorithms in malignant tachyarrhythmias. **Methods**: We retrospectively analyzed data from all patients with ICDs from a single manufacturer (Biotronik, Berlin, Germany) who were remotely monitored and followed up at two tertiary centers. Patients were divided into SC and DC groups, based on the programmed discrimination algorithm. The primary outcome was the risk of inappropriate therapies comparing SC vs. DC discriminators. A sensitivity analysis was also conducted, including only a subgroup of SC patients with active morphology discrimination. **Results**: A total of 557 patients were included. The distribution of the implanted ICDs was as follows: 76 VVI; 226 VDD; 76 DDD; and 179 CRT-D devices. A total of 124 ICDs were programmed utilizing SC and 433 were programmed into the DC discriminators group. Among the SC group, 47 (39%) ICDs used active morphology discrimination. The incidence of inappropriate ICD therapies did not differ among the SC and DC discrimination groups (Hazard Ratio [HR] 1.165; 95% Confidence Interval [CI] 0.393–3.448; *p* = 0.783). The predefined sensitivity analysis did not reveal any significant difference regarding this outcome (HR 1.809; 95% CI 0.241–13.577; *p* = 0.564). **Conclusions**: In this bicentric, remote monitoring-based study, the risk of inappropriate therapy in the SC group was similar to that of the DC group. Based on our results, SC discrimination is a suitable option, even for patients with dual-chamber devices.

## 1. Introduction

Implantable cardioverter defibrillators (ICDs) play an essential role in the prevention of sudden cardiac death [1]. Through the accurate detection and discrimination of tachyarrhythmias, ICDs can identify malignant ventricular arrhythmias and deliver appropriate therapy [1,2,3]. ICDs apply several tachyarrhythmia discrimination algorithms to differentiate between tachyarrhythmias of supraventricular and ventricular origin. These algorithms also play an important role in withholding therapy for supraventricular arrhythmias or in cases of oversensing of cardiac and non-cardiac signals (e.g., T-wave oversensing, electromagnetic interference, etc.) [2,3,4,5].

The two main configurations of ICDs are single-chamber (SC) and dual-chamber (DC) discrimination. While SC discriminators analyze only ventricular signals, DC discriminators analyze both atrial and ventricular electric activity and are also able to estimate atrioventricular (AV) synchrony [2,4,5,6]. Tachyarrhythmia discrimination is based on a broad scale of discriminator algorithms, with differences between devices depending on the device manufacturers and ICD programming [4,5,6,7,8,9,10].

Data in the literature comparing SC vs. DC arrhythmia discrimination are still limited and inconclusive [11]. Although some data support the superiority of DC algorithms, these mostly rely on previous generation devices from diverse manufacturers. Furthermore, morphology discrimination was only available in a small portion of SC ICD systems [12]. With the development of modern morphology discrimination algorithms, SC algorithms showed improvement in efficacy; non-inferiority was achieved in various reports [3,13,14]. As a consequence, an expert consensus statement on the optimal ICD programming and testing of HRS/EHRA/APHRS/SOLAECE (2015) stated that atrial lead should not be implanted solely for SVT discrimination [15]. Contrary to this, some recent reports indicate the superiority of DC algorithms [16,17].

Based on these controversial data, there is an obvious need for additional, manufacturer-specific comparisons of novel discrimination algorithms. The aim of this retrospective, bicentric study was to evaluate the efficacy of single- versus dual-chamber discrimination algorithms of ICD devices from a single manufacturer by performing a direct, head-to-head comparison, using data from a remote monitoring system. Our hypothesis was that SC discriminator algorithms, particularly those based on active morphological algorithms, exhibit comparable (or even superior) effectiveness when compared to DC discriminators.

## 2. Materials and Methods

### 2.1. Patient Population and Data Collection

Data were retrospectively collected from two tertiary referral centers (Cardiology Center, University of Szeged, Szeged, Hungary; Department of Cardiology, Central Hospital of Northern Pest–Military Hospital, Budapest, Hungary). All consecutive patients with an implanted ICD from a single manufacturer (Biotronik, Berlin, Germany), who were remotely followed up via the Home Monitoring^®^ (HM) system, were included. The registration dates in the HM system fell within the period from 2009 to 2024.

As baseline clinical characteristics, we collected the following data: the age at ICD implantation; time from ICD implantation to Home Monitoring registration; gender; ICD indication (i.e., primary or secondary); chronic coronary syndromes; structural heart diseases; indications for antibradycardia pacing; and applied tachyarrhythmia discrimination algorithms (i.e., SC or DC). Comorbidity data were also collected such as hypertension, previously diagnosed atrial fibrillation or atrial flutter, diabetes mellitus, and prior stroke or transient ischemic attack (TIA). Additionally, ECG parameters (heart rate, QRS morphology), left ventricular ejection fraction (LVEF), baseline laboratory values (creatinine level, estimated glomerular filtration rate (eGFR), hemoglobin level), baseline medical therapy, ICD sensing parameters at HM registration and pacing percentages at one month after HM registration were analyzed. Furthermore, we evaluated the baseline detection limits of the different VT zones. The study was approved by the Institutional Review Board (IRB) of the University of Szeged (No. 5514).

### 2.2. Study Endpoints

Patients were divided into two groups based on the applied discrimination algorithm: SC and DC discrimination groups. The primary outcome was the time to first inappropriate therapy, which was compared between the two patient groups. We also separately assessed first inappropriate therapies that resulted in antitachycardia pacing (ATP) delivery alone and therapies that resulted in ATP + shock delivery. Secondary endpoints included the time to first appropriate ICD therapy and all-cause mortality. Additionally, a sensitivity analysis was performed including only a subgroup of SC patients with active morphology discrimination to assess the risk of inappropriate therapy compared to the DC group. Finally, a subgroup analysis was also conducted within the DC group, comparing the incidence of inappropriate therapies between patients with VDD versus DDD devices.

To evaluate the appropriateness of the delivered ICD therapies, we reviewed the intracardiac electrograms (EGMs) recorded by the HM system. EGMs were adjudicated by an expert physician and a clinical field engineer, while uncertain cases were further evaluated by a senior electrophysiologist. Only episodes resulting in delivered ICD therapy (ATP and/or shock) were considered. If the discrimination algorithm was reprogrammed during follow-up—such as a switch between SC and DC configurations—patient data were analyzed only until the original settings remained unchanged.

### 2.3. Statistical Analysis

Statistical analysis was conducted using SPSS Statistics (version 29, IBM, Armonk, NY, USA) software. Continuous variables are presented as “median (first quartile (Q1)–third quartile (Q3))” forms and categorical variables as numbers (percentages). For the comparison of continuous variables, the Mann–Whitney U test was performed (due to non-normal distribution in all cases). For categorical variables, we conducted chi-squared tests.

In the primary analysis, we evaluated the risk of inappropriate ICD therapies, appropriate ICD therapies, and all-cause mortality between the SC and DC groups by conducting a time-to-event analysis calculating hazard ratios (HR) along with 95% confidence intervals (CI). Statistical significance was determined as a *p*-value ≤ 0.05. Similarly, to assess the risk of inappropriate therapies, the statistical method of time-to-event analysis was also used in the predefined sensitivity analysis and in the subgroup analysis of DC VDD vs. DC DDD ICDs. Time-to-event analysis was completed with a multivariate model in all the aforementioned cases. All predictor variables, which were considered potentially impactful regarding inappropriate therapies, appropriate therapies or all-cause mortality, underwent a univariate analysis. These variables comprised age at implantation, gender, primary prophylaxis, ischemic etiology, previously diagnosed atrial fibrillation or atrial flutter, hypertension, diabetes mellitus, stroke/TIA, bradypacing indication, baseline LVEF, heart rate, baseline creatinine, eGFR and hemoglobin levels, antiplatelet therapy, anticoagulation, therapy with beta-blocker, angiotensin-converting-enzyme inhibitor (ACEI)/angiotensin II receptor blocker (ARB)/angiotensin receptor-neprilysin inhibitor (ARNI), diuretic, mineralocorticoid receptor antagonist (MRA), digitalis glycoside, calcium channel blocker, amiodarone, statin, and sodium–glucose cotransporter-2 (SGLT-2) inhibitor. In the applied multivariate models, we included all predictor variables that demonstrated a statistically significant association (*p* ≤ 0.1) in the univariate analysis. The discrimination algorithm was included in the multivariate models regardless of the significance in the univariate analysis. Based on the same principle, device type was included in the multivariate model of the predefined subgroup. Lastly, we also separately assessed the risk of inappropriate ATP delivery alone and the risk of inappropriate ATP + shock delivery between SC and DC groups by performing time-to-event analysis.

## 3. Results

### 3.1. Baseline Clinical Characteristics

**Figure 1:**
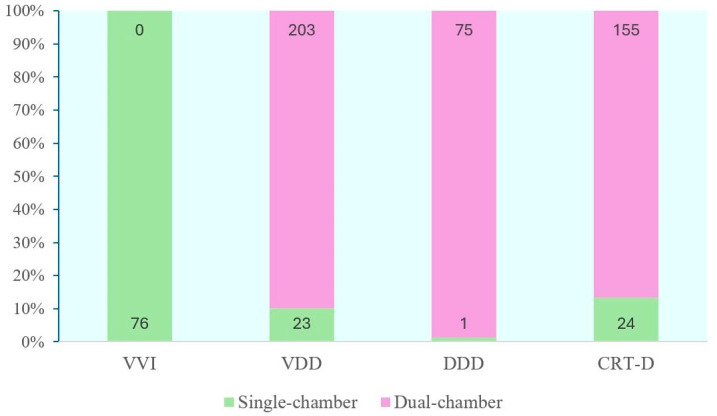
Single-chamber and dual-chamber discrimination algorithms by ICD device type. VVI: single-chamber ICD; VDD: single-lead ICD device with a floating atrial dipole; DDD: dual-chamber ICD; CRT-D: cardiac resynchronization therapy with defibrillator.

A total of 557 patients were included in the current study. Among them, 76 patients were implanted with a VVI, 226 with a VDD, and 76 with a DDD ICD device. A total of 179 received a CRT-D system (Table 1). The list of the implanted device models can be found in the Supplementary Materials (Table S1). Of the total cohort, 124 patients were assigned to the SC discrimination and 433 patients were assigned to the DC group. The morphology discrimination algorithm (i.e., MorphMatch) was active in 39% of patients in the SC group (n = 47). The distribution of discrimination algorithms across the different types of ICD is presented in Figure 1.

The median age at ICD implantation was 65 (55–72) years and 77% of the patients were male. ICD implantation was primary prophylactic in 58%. Half of the patients (49%) suffered from ischemic heart disease and 39% were previously diagnosed with atrial fibrillation or atrial flutter. The detailed comparison of baseline clinical characteristics between the SC and DC discriminator group is presented in Table 1. Notably, patients in the SC group were older at the time of device implantation (*p* = 0.002). The primary prophylaxis was more common in the DC group (61% vs. 46%), whereas a history of atrial fibrillation or flutter was significantly more prevalent in the SC group (58% vs. 33%, *p* < 0.001).

With respect to major cardiovascular risk factors, the prevalence of hypertension was significantly higher in the SC group (89% vs. 76%, *p* = 0.002), while the incidence of diabetes mellitus and prior stroke/TIA did not differ between the groups. Although the overall indication for bradypacing was comparable, the distribution of underlying conduction disorders varied; atrioventricular (AV) block was more frequently observed in the SC group, whereas sick sinus syndrome was more prevalent in the DC group. Furthermore, patients in the SC group had a higher baseline left ventricular ejection fraction (median 35% vs. 30%, *p* = 0.002). Baseline ECG parameters and laboratory values showed no differences between the SC and DC groups.

Baseline medical therapy was generally comparable between the SC and DC discriminator groups, with the exception of diuretics and mineralocorticoid receptor antagonists (MRA), which were more frequently administered in the DC group (70% vs. 60%, *p* = 0.030; and 74% vs. 61%, *p* = 0.003, respectively) (Table 2).

### 3.2. Sensing/Pacing Parameters and VT Zone Settings

At the time of HM registration, atrial sensing values were comparable between the SC and DC discrimination groups (5.7 [3.4–6.8] mV vs. 4.1 [2.4–6.3] mV; *p* = 0.166) (Figure S1A), whereas ventricular sensing was higher in the DC group (14.6 [9.4–19.5] mV vs. 16.9 [12.0–20.0] mV; *p* = 0.016) (Figure S1B). Ventricular pacing percentages were also significantly higher in the DC group compared to the SC group (0 [0–21]% vs. 1 [0–96]%; *p* = 0.002) (Table 3).

The baseline lower detection limit for the VT1 zone did not differ between the SC and DC groups (340 [330–375] ms vs. 340 [330–360] ms). The lower limit for the VT2 zone was slightly higher in the DC group (320 [290–340] ms vs. 300 [290–320] ms). For the VF zone, the lower limit was similar: 260 [253–280] ms in the SC and 260 [250–280] ms in the DC group.

### 3.3. Clinical Outcomes

**Figure 2:**
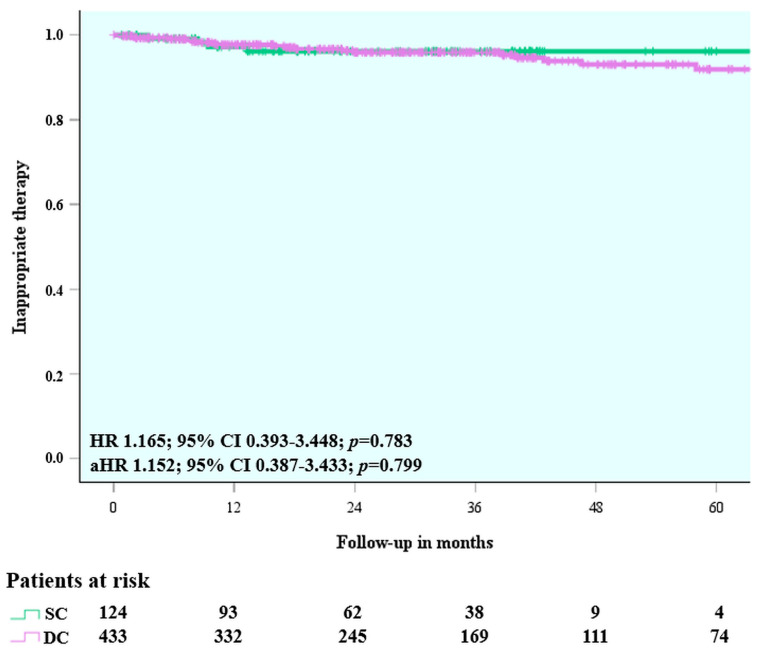
Time to first inappropriate therapy—single-chamber vs. dual-chamber. HR: hazard ratio; aHR: adjusted hazard ratio; CI: confidence interval; SC: single-chamber; DC: dual-chamber.

The median follow-up time was 2.4 (1.1–3.6) years. There was only one case when the end of the assessed follow-up was a switch between discrimination algorithms. In this case, DC discrimination algorithm was changed to SC algorithm due to inappropriate therapy delivery to sinus tachycardia caused by atrial undersensing. The incidence of inappropriate therapies was 3.2% (0.01% per patient-year) in the SC and 4.4% (0.01% per patient-year) in the DC discrimination group. Time-to-event analysis showed no difference between the patient groups (HR 1.165; 95% CI 0.393–3.448; *p* = 0.783; adjusted HR 1.152; 95% CI 0.387–3.433; *p* = 0.799) (Table 4, Figure 2, Table S2). Further analysis of inappropriate therapies resulted in ATP delivery alone (HR 1.264; 95% CI 0.365–4.377; *p* = 0.712) (Table 4) and inappropriate therapies resulted in ATP + shock delivery (HR 0.871; 95% CI 0.091–8.372; *p* = 0.905) (Table 4) revealed no difference between the SC and DC discrimination groups. Furthermore, in the predefined sensitivity analysis comparing the SC algorithm with activated morphology discrimination to the DC algorithm, no difference was observed in the risk of inappropriate therapies (HR 1.809; 95% CI 0.241–13.577; *p* = 0.564; adjusted HR 1.571; 95% CI 0.208–11.851; *p* = 0.661) (Table 4, Table S3, Figure S2). The specific reasons of inappropriate therapy delivery are summarized in Table 5. In the predefined subgroup analysis, no difference was observed in the risk of inappropriate therapies between patients with VDD devices and those with DDD devices, both using DC discrimination (HR 0.586; 95% CI 0.230–1.490; *p* = 0.262; adjusted HR 0.597; 95% CI 0.226–1.579; *p* = 0.299) (Table 4, Table S4, Figure S3).

Similarly, the rate of appropriate therapies did not differ between the SC and DC discrimination groups (15.3% (0.07% per patient-year) vs. 12.9% (0.04% per patient-year)) (HR 0.724; 95% CI 0.428–1.224; *p* = 0.228; adjusted HR 0.699; 95% CI 0.389–1.257; *p* = 0.232) (Table 4 and, Table S5, Figure S4).

All-cause mortality was also similar in both SC and DC discriminator groups (21.6% (0.09% per patient-year) vs. 26.6% (0.09% per patient-year)) (HR 0.930; 95% CI 0.598–1.448; *p* = 0.749; adjusted HR 0.714; 95% CI 0.426–1.197; *p* = 0.201) (Table 4, Table S6, Figure S5).

## 4. Discussion

### 4.1. Main Findings

In this bicentric cohort study, we conducted a head-to-head comparison of SC and DC discrimination algorithms using remote monitoring data, focusing exclusively on devices from a single manufacturer. Our aim was to evaluate the feasibility and efficacy of SC discrimination in comparison to DC discrimination for tachyarrhythmia detection. The results demonstrated that SC discrimination is non-inferior to DC algorithms in this context. To the best of our knowledge, this is the first study that directly compare SC and DC discrimination strategies specifically in single-manufacturer devices. Additionally, we performed a sensitivity analysis to evaluate the role of active morphology discrimination within SC devices. Our findings provide further evidence supporting the first-line use of single-chamber discrimination with active morphology analysis even in dual-chamber ICDs.

### 4.2. Tachyarrhythmia Discrimination

The primary components of tachyarrhythmia discrimination can differ significantly between device manufacturers [2,4,8]. Modern Biotronik devices rely primarily on stability, sudden onset, and morphology analysis of ventricular signals (“MorphMatch algorithm”) for SC discrimination. In contrast, the DC discrimination algorithm (i.e., SMART detection) can detect atrial signals, enabling additional discrimination methods such as the comparison of atrial and ventricular frequencies and algorithms analyzing the atrioventricular synchrony (AV-trend, AV-regularity). Notably, in Biotronik ICDs, DC configuration does not incorporate morphology-based differentiation, unlike other manufacturers [4,5,6,9,10]. A recent study by Biffi et al. compared SC and DC discrimination algorithms in single-chamber devices. In that study, the SC group included conventional single-lead ICDs from three manufacturers (Medtronic, Boston Scientific and Abbott); most were equipped with active morphology discrimination. The DC group consisted exclusively of Biotronik VDD ICDs (single-lead ICDs with a floating atrial dipole) programmed with the SMART detection algorithm. The study found no significant difference in the tachyarrhythmia discrimination efficacy between SC and DC groups (HR 0.81; 95% CI 0.38–1.72; *p* = 0.586) [3]. It is important to note that earlier studies including implantable cardioverter defibrillators from Biotronik also involved ICDs from other manufacturers and that morphology discrimination (MorphMatch) was not an available option at that time [12,18,19].

### 4.3. Possible Causes of Inappropriate Detection in SC and DC Configurations

Based on the major differences in tachyarrhythmia discrimination among manufacturers, the role of single-manufacturer studies should be emphasized, given the fact that SC and DC discrimination algorithms are both programmable options in dual-chamber devices [11]. The latest update of the expert consensus statement of HRS/EHRA/APHRS/LAHRS (2019) suggests the programming of SC vs. DC discrimination algorithms according to the number of implanted leads [8]. However, there are scenarios when DC discrimination may fail and result in inappropriate therapy delivery and that switching to SC discrimination can provide a reasonable solution. Conversely, the opposite scenario is also conceivable: following an inappropriate shock under SC discrimination, a switch to DC discrimination might be required to ensure more accurate rhythm classification.

In our bicentric cohort, all inappropriate therapies were primarily associated with supraventricular arrhythmias (Table 5). The most common reasons were regular supraventricular tachycardias (SVTs) with a 1:1 AV ratio (sinus tachycardia or paroxysmal supraventricular tachycardia (PSVT)). The second most frequent underlying rhythm was atrial fibrillation (and atrial flutter). Notably, the mechanism of these inadequate detections varied among these cases.

**Figure 3:**
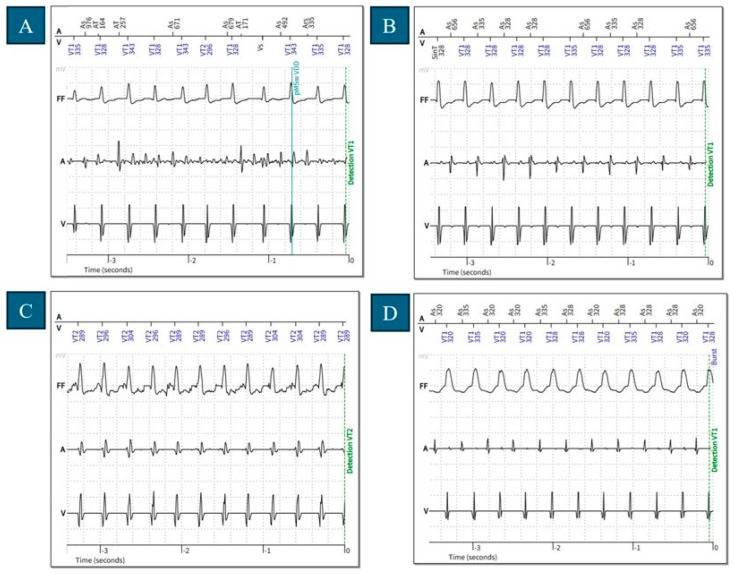
Main reasons leading to inappropriate detection in cases of dual-chamber discrimination: (**A**) intermittent atrial undersensing during atrial fibrillation (last measured RA sensing value: 3.2 mV; device type: Iforia 5 VR-T DX); (**B**) atrial undersensing during sinus tachycardia (last measured RA sensing value: 3.2 mV; device type: Iforia 5 VR-T DX); and (**C**) inappropriate VT detection due to blanking of the atrial signals (last measured RA sensing value: 2.8 mV; device type: Itrevia 5 VR-T DX). (**D**) 1:1 SVT with stable RR and PP intervals. The sudden onset criterium was fulfilled leading to inappropriate VT detection (last measured RA sensing value: 2.4 mV; device type: Lumax 640 DR-T). All the cases resulted in inappropriate therapy delivery. VT: ventricular tachycardia; SVT: supraventricular tachycardia; A: atrial electrogram; V: ventricular electrogram; FF: far-field electrogram; RA: right atrium; VDD: single-lead ICD device with a floating atrial dipole; DDD: dual-chamber ICD,;VT1: lower-rate ventricular tachycardia; VT2: higher-rate ventricular tachycardia; As: atrial sensed event; AT: atrial tachycardia; Vs: ventricular sensed event; Ars: atrial refractory sensed event; pMSw: pacing mode switch; SinT: sinus tachycardia.

In the DC discrimination group, most inappropriate therapy delivery resulted from inadequate detection or the misinterpretation of atrial signals. The initial step in tachycardia discrimination with SMART is the frequency comparison of ventricular and atrial signals [2,4,5]. True atrial undersensing or atrial blanking can lead the device to underestimate the atrial rate, causing a false interpretation of “higher ventricular than atrial frequency”, which triggers VT detection and consequently therapy delivery [4] (Figure 3A,B). Although atrial lead (or sensing dipole) dysfunction can cause atrial undersensing, atrial fibrillation itself may lead to intermittently low signal amplitudes—meaning that atrial undersensing during atrial fibrillation does not necessarily indicate electrode malfunction. Notably, in our study, high atrial signal amplitudes in both VDD and DDD ICD recipients were observed (Figure S1A). Both our results and those from previous studies in the literature suggest that the arrhythmia discrimination capability of VDD ICDs is non-inferior to that of DDD systems [20,21,22].

Atrial blanking was also associated with inappropriate therapy delivery in two cases. In Biotronik devices, the far-field protection mechanism triggers an atrial blanking period around each ventricular sensed event, during which atrial signals are ignored in tachycardia discrimination. The blanking lasts for a fixed value of 16 ms before each ventricular sensed event and the total blanking duration is programmable (default 75 ms). This may lead to inaccurate atrial–ventricular rate comparison and inappropriate VT detection [5] (Figure 3C). Interestingly, we also identified cases where atrial undersensing occurred outside the atrial blanking during sinus tachycardia (Table 5, Figure 3B). In these cases, undersensing was connected to the fluctuation of right atrial sensing values, which may be associated with suboptimal lead positioning.

Finally, during tachyarrhythmias characterized by equal and stable PP and RR intervals, as well as regular AV times, the SMART algorithm uses the sudden onset discriminator to differentiate between supraventricular or ventricular origin: sudden onset indicates VT, while gradual onset indicates sinus tachycardia [4,5]. However, PSVT episodes or atrial runs/ectopic atrial rhythms (unlike sinus tachycardia) can also meet these criteria, potentially misleading the onset discriminator and resulting in inappropriate VT detection (Figure 3D).

In these aforementioned scenarios, opting for SC discrimination could be a reasonable solution, as it addresses issues related to inappropriate atrial detection (i.e., atrial undersensing) by relying solely on ventricular signals aided by morphology-based algorithms. Furthermore, Safak et al. previously described cases where SMART detection misclassified actual VT episodes as SVTs, leading to the omission of therapy [23]. It should be noted, however, that DC discriminators remain under continuous development. For instance, an updated version of the SMART algorithm has been introduced that addresses false atrial undersensing caused by far-field protection via reducing the atrial blanking period duration from 75 ms to 25 ms if the ventricular rate enters the VT zone [24].

In the SC group, most inappropriate therapies occurred in cases where the MorphMatch algorithm was not available; discrimination relied solely on stability and sudden onset criteria (Table 5). Notably, MorphMatch is a relatively recent technology developed in the 2010s. The latest update of the expert consensus statement of HRS/EHRA/APHRS/LAHRS (2019) recommends programming the MorphMatch algorithm without stability and sudden onset in SC devices [8].

However, morphology-based discrimination should be used with caution in certain scenarios. The algorithm compares a continuously updated baseline template to ongoing QRS morphologies, with significant deviations indicating VT [4,5]. Nonetheless, rate-dependent bundle branch block or aberrantly conducted SVTs may mimic VT [2,4,5]. When stable templates cannot be obtained—such as in cases of complete AV block or biventricular pacing—MorphMatch is not recommended [4,24].

**Figure 4:**
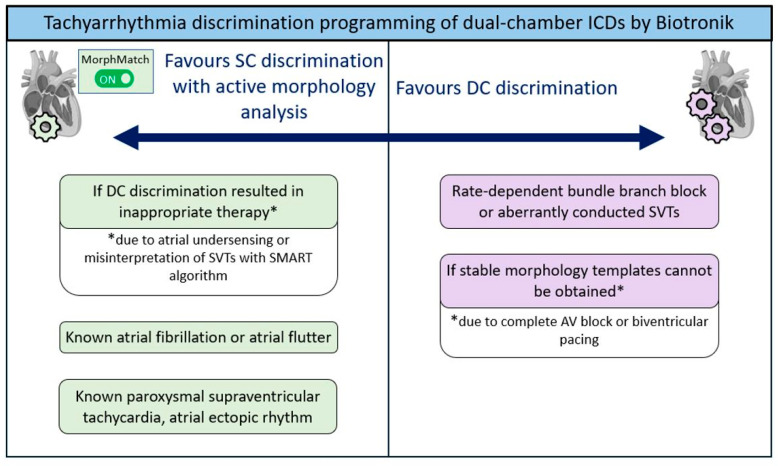
Recommendations for tachyarrhythmia discrimination programming of dual-chamber devices (i.e., VDD or DDD ICDs) by Biotronik. * Represents additional details described in the white boxes underneath. SC: single-chamber, DC: dual-chamber, ICD: implantable cardioverter defibrillator, SMART: specific morphology-based arrhythmia recognition technology, SVT: supraventricular tachycardia. SC discrimination with active morphology analysis appears to be a preferable option if former DC discrimination resulted in inappropriate therapy delivery due to inappropriate atrial sensing (e.g., atrial undersensing) or misinterpretation of SVTs caused by false decisions of the SMART algorithm. Furthermore, SC discrimination with active morphology analysis could be a reasonable first-line option in patients with known supraventricular arrhythmias (e.g., atrial fibrillation, atrial flutter, paroxysmal supraventricular tachycardia, atrial ectopic rhythm). However, DC discrimination should be preferred in patients with rate-dependent bundle branch block or previously documented SVT with aberrant conduction. Lastly, SC discrimination should be avoided in patients where stable morphology templates cannot be obtained (e.g., complete AV block or biventricular pacing).

Although, our study demonstrated only the non-inferiority of SC discriminators compared to DC algorithms—and did not confirm superiority- the rate of inappropriate therapies was numerically lower with SC discrimination. Regarding non-inferiority, these findings are aligned with the results of Biffi et al. [3]. These, along with the theoretical considerations discussed earlier, suggest a potential clinical advantage of SC algorithms in most cases. However, confirming superiority would require larger, adequately powered studies. Furthermore, based on our results, in cases of atrial electrode failure, the replacement of the impaired electrode can be avoided if the patient does not require atrial pacing and the indication for replacement is limited to the DC discrimination algorithm. Additionally, the availability of DC tachycardia discrimination alone does not justify atrial electrode implantation. A brief summary of our recommendations for discrimination programming in dual-chamber devices is shown in Figure 4, integrating our empirical findings and the current body of evidence.

Finally, although the benefits of atrial sensing in tachyarrhythmia discrimination may be questionable in certain contexts, its role in atrial fibrillation detection should not be underestimated. As demonstrated in various previous studies, the primary advantage of atrial sensing is the enhanced ability to identify atrial arrhythmias that can aid optimal patient management in atrial fibrillation. In this context, the clinical significance of VDD ICDs is particularly noteworthy, as these systems offer reliable atrial sensing to detect atrial high-rate episodes with just a single lead implantation [20,21,25].

### 4.4. Study Limitations

Data were analyzed retrospectively; therefore, all potential limitations of such a design apply, including the limited availability of detailed data regarding causes of death. Notably, baseline differences existed between the SC and DC discrimination groups. As the ejection fraction was significantly lower in the DC discrimination group, the primary prophylactic indication for ICD implantation and the indication for CRT devices were more frequently observed, accompanied by the higher rates of MRA and diuretic therapy. Moreover, the rate of previously diagnosed atrial fibrillation/atrial flutter was lower in the DC group. However, the higher risk for supraventricular tachyarrhythmias in the SC groups supports the non-inferiority of SC discrimination algorithms, as high-frequency supraventricular arrhythmias are one of the most frequent causes of inappropriate therapy delivery. Nevertheless, the core of tachyarrhythmia discrimination was the interpretation of intracardiac EGMs; sensing quality was high and adequate in both groups. Notably, our study also included older devices (from 2009), in which morphology discrimination was not universally available. Finally, the incidence of inappropriate therapies was infrequent during a relatively short follow-up period.

## 5. Conclusions

Single-chamber discrimination proved to be non-inferior to dual-chamber algorithms in detecting malignant tachyarrhythmias. Considering its comparable performance and potential clinical advantages—particularly when an active morphology-based discriminator is available—SC discrimination may represent a valid and efficient programming option in Biotronik ICDs, even in patients implanted with dual-chamber devices.

## Figures and Tables

**Table 1:** Baseline clinical characteristics.

	Overall (N = 557)	Single-Chamber (N = 124)	Dual-Chamber (N = 433)	*p*-Value
Age at implantation, years (median [Q1–Q3])	65 (55–72)	67 (59–75)	64 (54–71)	**0.002**
Time from ICD implantation to Home Monitoring registration, days (median [Q1–Q3])	5 (1–225)	129 (3–682)	4 (1–62)	**<0.001**
Male, n (%)	431 (77%)	94 (76%)	337 (78%)	0.635
Primary prophylaxis, n (%) ^a^	321 (58%)	57 (46%)	264 (61%)	**0.002**
Chronic coronary syndromes, n (%) ^b^	270 (49%)	61 (49%)	209 (49%)	0.979
Structural heart disease, n (%) ^c^	460 (83%)	99 (80%)	361 (84%)	0.282
*Ischemic cardiomyopathy*	266 (48%)	61 (49%)	205 (48%)	
*Dilatative cardiomyopathy*	165 (30%)	32 (26%)	133 (31%)	
*Hypertrophic cardiomyopathy*	23 (4%)	6 (5%)	17 (4%)	
*Arrhythmogenic right ventricular cardiomyopathy*	6 (1%)	0 (0%)	6 (1%)	
*Other*	94 (17%)	25 (20%)	69 (16%)	
Previously diagnosed atrial fibrillation/atrial flutter, n (%) ^c^	213 (39%)	71 (58 %)	142 (33%)	**<0.001**
*Paroxysmal*	117 (55%)	21 (29.5%)	96 (67.5%)	
*Persistent*	23 (11%)	2 (3%)	21 (15%)	
*Permanent*	73 (34%)	48 (67.5%)	25 (17.5%)	
Hypertension, n (%) ^a^	436 (79%)	110 (89%)	326 (76%)	**0.002**
Diabetes mellitus, n (%) ^a^	166 (30%)	39 (32%)	127 (30%)	0.670
Stroke/TIA, n (%) ^c^	39 (7%)	13 (11%)	26 (6%)	0.083
Bradypacing indication, n (%) ^d^	98 (18%)	20 (16%)	78 (18%)	0.623
No bradypacing indication	454 (82%)	103 (84%)	351 (82%)	
Sick sinus syndrome	44 (8%)	2 (2%)	42 (10%)	
AV block in sinus rhythm	34 (6%)	3 (2%)	31 (7%)	
Atrial fibrillation with slow ventricular response	20 (4%)	15 (12%)	5 (1%)	
QRS, n (%) ^e^				0.545
*Narrow QRS*	255 (48%)	57 (50%)	198 (47%)	
*LBBB*	115 (21%)	20 (17%)	95 (22%)	
*RBBB*	43 (8%)	10 (9%)	33 (8%)	
*Paced rhythm/Other*	124 (23%)	27 (24%)	97 (23%)	
LVEF % (median [Q1–Q3]) ^f^	30 (25–40)	35 (25–48)	30 (23–38)	**0.002**
Heart rate, bpm (median [Q1–Q3]) ^g^	70 (61–81)	73 (62–84)	70 (60–81)	0.273
Creatinine, umol/L (median [Q1–Q3]) ^h^	98 (82–120)	99 (80–119)	98 (82–121)	0.615
eGFR, mL/min/1.73 m^2^ (median [Q1–Q3]) ^i^	63 (50–81)	60 (48–80)	64 (50–81)	0.473
Hemoglobin, mmol/L (median [Q1–Q3]) ^j^	137 (125–148)	135 (121–148)	137 (125–148)	0.449
Implanted device type, n (%)				**<0.001**
*VVI*	76 (14%)	76 (61%)	0 (0%)	
*VDD*	226 (40%)	23 (19%)	203 (47%)	
*DDD*	76 (14%)	1 (<1%)	75 (17%)	
*CRT-D* ^k^	179 (32%)	24 (19%)	155 (36%)	

^a^ Available for 555 patients. ^b^ Available for 550 patients. ^c^ Available for 554 patients. ^d^ Available for 552 patients. ^e^ Available for 537 patients. ^f^ Available for 541 patients. ^g^ Available for 539 patients. ^h^ Available for 519 patients. ^i^ Available for 500 patients. ^j^ Available for 517 patients. ^k^ In the SC discrimination group, 20 CRT-D devices were implanted without an atrial lead. TIA: transient ischemic attack; AV: atrioventricular; LBBB: left bundle branch block; RBBB: right bundle branch block; LVEF: left ventricular ejection fraction; eGFR: estimated glomerular filtration rate; VVI: single-chamber ICD; VDD: single-lead ICD device with a floating atrial dipole; DDD: dual-chamber ICD; CRT-D: cardiac resynchronization therapy with defibrillator.

**Table 2:** Baseline medical therapy.

	Overall (N = 557)	Single-Chamber (N = 124)	Dual-Chamber (N = 433)	*p*-Value
Antiplatelets, n (%) ^a^	266 (48%)	51 (41%)	215 (50%)	0.082
Anticoagulation, n (%) ^a^	311 (56%)	77 (62%)	234 (54%)	0.129
Beta-blocker, n (%) ^a^	534 (96%)	120 (97%)	414 (96%)	0.795
RAAS inhibitor, n (%) ^b^	493 (89%)	108 (88%)	385 (90%)	0.586
Diuretics, n (%) ^a^	375 (68%)	74 (60%)	301 (70%)	**0.030**
MRA, n (%) ^a^	395 (71%)	75 (61%)	320 (74%)	**0.003**
Digitalis, n (%) ^a^	35 (6%)	11 (9%)	24 (6%)	0.185
CCB, n (%) ^a^	54 (10%)	17 (14%)	37 (9%)	0.091
Amiodaron, n (%) ^a^	144 (26%)	28 (23%)	116 (27%)	0.325
Statin, n (%) ^a^	364 (66%)	80 (65%)	284 (66%)	0.752
SGLT2-inhibitor, n (%) ^b^	87 (16%)	20 (16%)	67 (16%)	0.890

^a^ Available for 554 patients. ^b^ Available for 553 patients. RAAS: renin–angiotensin–aldosterone system; MRA: mineralocorticoid receptor antagonist; CCB: calcium channel blocker; SGLT2-inhibitor: sodium–glucose cotransporter-2.

**Table 3:** Baseline sensing/pacing parameters.

	Single-Chamber (N = 124)	Dual-Chamber (N = 433)	*p*-Value
Atrial sensing at HM registration, mV (median [Q1–Q3]) ^a^	5.7 (3.4–6.8)	4.1 (2.4–6.3)	0.166
Ventricular sensing at HM registration, mV (median [Q1–Q3]) ^b^	14.6 (9.4–19.5)	16.9 (12.0–20.0)	**0.016**
Atrial pacing at 1st month after HM registration, % (median [Q1–Q3]) ^c^	n/a	3.0 (0.0–42.0)	
Ventricular pacing at 1st month after HM registration, % (median [Q1–Q3]) ^d^	0 (0–21)	1 (0–96)	**0.002**

^a^ Available for 428 patients. ^b^ Available for 515 patients. ^c^ Available for 224 patients. ^d^ Available for 544 patients. HM: Home Monitoring; n/a: not applicable.

**Table 4:** Clinical outcomes.

	Single-Chamber (N = 124)	Dual-Chamber (N = 433)	*p*-Value
Inappropriate therapy, n (%)	4 (3.2%)	19 (4.4%)	0.566
*resulted in ATP therapy alone*	3 (75%)	16 (84%)	0.659
*resulted in ATP + shock therapy*	1 (25%)	3 (16%)	
Appropriate therapy, n (%)	19 (15.3%)	56 (12.9%)	0.492
	**Single-chamber vs. Dual-chamber**	**95% CI**	***p*** **-value**
Inappropriate therapy	HR (univariate) 1.165	0.393–3.448	0.783
	HR (multivariate) ^a^ 1.152	0.387–3.433	0.799
*resulted in ATP therapy alone*	HR (univariate) 1.264	0.365–4.377	0.712
*resulted in ATP + shock therapy*	HR (univariate) 0.871	0.091–8.372	0.905
Appropriate therapy	HR (univariate) 0.724	0.428–1.224	0.228
	HR (multivariate) ^b^ 0.699	0.389–1.257	0.232
All-cause mortality	HR (univariate) 0.930	0.598–1.448	0.749
	HR (multivariate) ^c^ 0.714	0.426–1.197	0.201
	**Single-chamber MorphMatch ON vs. Dual-chamber**		
Inappropriate therapy	HR (univariate) 1.809	0.241–13.577	0.564
	HR (multivariate) ^d^ 1.571	0.208–11.851	0.661
	**Dual-chamber VDD vs. Dual-chamber DDD**		
Inappropriate therapy	HR (univariate) 0.586	0.230–1.490	0.262
	HR (multivariate) ^e^ 0.597	0.226–1.579	0.299

^a^ Available for 553 patients. ^b^ Available for 509 patients. ^c^ Available for 467 patients. ^d^ Available for 476 patients. ^e^ Available for 423 patients. ATP: antitachycardia pacing; HR: hazard ratio; CI: confidence interval; VDD: single-lead ICD device with a floating atrial dipole; DDD: dual-chamber ICD.

**Table 5:** Specific causes of inappropriate therapy delivery by the programmed discrimination algorithms.

	Single-Chamber (N = 124)	Dual-Chamber (N = 433)
Underlying arrhythmia, n		
Atrial fibrillation or atrial flutter	3	5
Sinus tachycardia or PSVT	1	14
Underlying mechanism, n *Atrial undersensing* *Atrial blanking causing misdetection of atrial* *rate by the SMART algorithm* *1:1 SVT, but the SMART algorithm identifies* *VT due to sudden onset* *Sinus tachycardia, but SC discrimination* *identifies VT due to sudden onset* *(MorphMatch algorithm was not available)* *Atrial fibrillation, but both stability and* *sudden onset identified VT (MorphMatch* *algorithm was not available)* *Other*	- - - 1 2 1	10 2 6 - - 1

PSVT: paroxysmal supraventricular tachycardia; SVT: supraventricular tachycardia; VT: ventricular tachycardia.

## Data Availability

All data used in this study are available by reasonable request from the corresponding author.

## Supplementary Materials

The following supporting information can be downloaded at: https://www.mdpi.com/article/10.3390/jcm14165859/s1, Table S1: list of implanted device models; Table S2: multivariate analysis of time to first inappropriate therapy—single-chamber vs. dual-chamber; Table S3: multivariate analysis of time to first inappropriate therapy—single-chamber (MorphMatch ON) vs. dual-chamber; Table S4: multivariate analysis of time to first inappropriate therapy—dual-chamber discriminator VDD vs. dual-chamber discriminator DDD; Table S5: multivariate analysis of time to first appropriate therapy—single-chamber vs. dual-chamber; Table S6: multivariate analysis of all-cause mortality—single-chamber vs. dual-chamber; Figure S1: (A) baseline atrial sensing parameters in single-chamber and dual-chamber discrimination groups; (B) baseline ventricular sensing parameters in single-chamber and dual-chamber discrimination groups; Figure S2: time to first inappropriate therapy—single-chamber (MorphMatch ON) vs. dual-chamber; Figure S3: time to first inappropriate therapy—dual-chamber discriminator VDD vs. dual-chamber discriminator DDD; Figure S4: time to first appropriate therapy—single-chamber vs. dual-chamber; Figure S5: all-cause mortality—single-chamber vs. dual-chamber.

## Abbreviations

The following abbreviations are used in this manuscript:

ACEI	angiotensin-converting-enzyme inhibitor
ARB	angiotensin II receptor blocker
ARNI	angiotensin receptor-neprilysin inhibitor
ARVC	arrhythmogenic right ventricular cardiomyopathy
ATP	antitachycardia pacing
AV	atrioventricular
CCB	calcium channel blocker
CI	confidence interval
CMP	cardiomyopathy
CRT	cardiac resynchronization therapy
CRT-D	cardiac resynchronization therapy with defibrillator
DC	dual-chamber
DDD ICD	dual-chamber ICD
eGFR	estimated glomerular filtration rate
EGM	electrogram
HCM	hypertrophic cardiomyopathy
HM	Home Monitoring
HR	hazard ratio
ICD	implantable cardioverter defibrillator
LBBB	left bundle branch block
LVEF	left ventricular ejection fraction
MRA	mineralocorticoid receptor antagonist
PSVT	paroxysmal supraventricular tachycardia
Q1	first quartile
Q3	third quartile
RAAS	renin-angiotensin-aldosterone system
RBBB	right bundle branch block
SC	single-chamber
SD	standard deviation
SGLT2	sodium–glucose cotransporter-2
SMART	specific, morphology-based arrhythmia recognition technology
SSS	sick sinus syndrome
SVT	supraventricular tachycardia
TIA	transient ischemic attack
VDD ICD	single-lead ICD device with a floating atrial dipole
VF	ventricular fibrillation
VT	ventricular tachycardia
VVI ICD	single-chamber ICD
