# Supplementary material for: Head-to-Head Comparison of Single- Versus Dual-Chamber ICD Discriminators for Tachyarrhythmia Detection: A Single-Manufacturer, Remote Monitoring-Based Bicentric Study
Source: J Clin Med. 2025 Aug 19;14(16):5859. doi: 10.3390/jcm14165859 (PMC12387967; doi:10.3390/jcm14165859)
Supplement: Supplementary file 1 [file jcm-14-05859-s001.zip › jcm-3778896-supplementary.pdf]

Journal of
Clinical Medicine

MDPI

Head-to-head comparison of single- versus dual-chamber ICD discriminators for tachyarrhythmia detection: a single-manufacturer, remote monitoring based, bicentric study

Supplementary material

Table S1. List of implanted device models

**List of implanted Biotronik device models, n (%)**			
Acticor 7 VR-T DX	1 (0.2%)	Itrevia 5 HF-T QP	1 (0.2%)
Iforia 5 DR-T	11 (2%)	Itrevia 5 VR-T DX	44 (7.9%)
Iforia 5 HF-T	10 (1.8%)	Itrevia 7 DR-T	6 (1.1%)
Iforia 5 VR-T DX	37 (6.6%)	Itrevia 7 HF-T	18 (3.2%)
Inlexa 3 VR-T	54 (9.7%)	Lumax 300 HF-T	8 (1.4%)
Intica 5 DR-T	1 (0.2%)	Lumax 340 HF-T	1 (0.2%)
Intica 5 VR-T DX	51 (9.1%)	Lumax 540 HF-T	13 (2.3%)
Intica 7 DR-T	25 (4.5%)	Lumax 540 VR-T DX	13 (2.3%)
Intica 7 HF-T	26 (4.7%)	Lumax 640 DR-T	4 (0.7%)
Intica 7 HF-T QP	20 (3.6%)	Lumax 640 HF-T	1 (0.2%)
Intica Neo 5 VR-T	2 (0.4%)	Lumax 640 VR-T DX	17 (3.1%)
Intica Neo 5 VR-T DX	23 (4.1%)	Rivacor 5 VR-T	20 (3.6%)
Intica Neo 7 DR-T	8 (1.4%)	Rivacor 5 VR-T DX	38 (6.8%)
Intica Neo 7 HF-T	16 (2.9%)	Rivacor 7 DR-T	21 (3.8%)
Intica Neo 7 HF-T QP	16 (2.9%)	Rivacor 7 HF-T	13 (2.3%)
Itrevia 5 HF-T	3 (0.5%)	Rivacor 7 HF-T QP	35 (6.3%)

Journal of Clinical Medicine

MDPI

Table S2. Multivariate analysis of time to first inappropriate therapy – single-chamber vs. dual-chamber.

Risk factor	unadjusted HR (95% CI)	p-value	adjusted HR (95% CI)	p-value
Discrimination algorythm	1.165 (0.393-3.448)	0.783	1.152 (0.387-3.433)	0.799
Age at implantation	0.988 (0.958-1.020)	0.462		
Male	1.109 (0.411-2.989)	0.838		
Secondary prophylaxis	1.957 (0.835-4.585)	0.122		
Ischemic etiology	0.608 (0.255-1.451)	0.262		
Previously diagnosed atrial fibrillation/atrial flutter	1.303 (0.555-3.059)	0.543		
Hypertension	0.800 (0.312-2.051)	0.643		
Diabetes mellitus	0.768 (0.283-2.085)	0.605		
Stroke/TIA	0.690 (0.093-5.136)	0.717		
Bradypacing indication	0.479 (0.112-2.049)	0.321		
LVEF	0.981 (0.947-1.016)	0.285		
Heart rate	0.994 (0.970-1.019)	0.638		
Creatinine	1.002 (0.995-1.010)	0.539		
eGFR	1.006 (0.984-1.030)	0.578		
Hemoglobin	1.000 (0.976-1.025)	0.993		
Antiplatelets	0.844 (0.364-1.954)	0.692		
Anticoagulation	1.048 (0.452-2.427)	0.913		
Beta-blocker	0.621 (0.083-4.629)	0.642		
RAAS	2.235 (0.300-16.632)	0.432		
Diuretics	0.704 (0.301-1.648)	0.419		
MRA	0.620 (0.265-1.454)	0.272		
Digitalis	1.618 (0.378-6.925)	0.516		
CCB	0.945 (0.221-4.049)	0.940		
Amiodaron	0.294 (0.069-1.257)	**0.099**	0.294 (0.069-1.257)	0.099
Statins	0.648 (0.280-1.500)	0.311		
SGLT2-inhibitors	0.041 (0.000-30.458)	0.344		

HR: hazard ratio, CI: confidence interval, TIA: transient ischemic attack, LVEF: left ventricular ejection fraction, eGFR: estimated glomerular filtration rate, RAAS: renin-angiotensin-aldosterone system, MRA: mineralocorticoid receptor antagonist, CCB: calcium channel blocker, SGLT2-inhibitor: sodium-glucose cotransporter-2.

Journal of
Clinical Medicine

Table S3. Multivariate analysis of time to first inappropriate therapy – single-chamber (MorphMatch ON) vs. dual-chamber.

Risk factor	unadjusted HR (95% CI)	p-value	adjusted HR (95% CI)	p-value
Discrimination algorythm	1.809 (0.241-13.577)	0.564	1.571 (0.208-11.851)	0.661
Age at implantation	0.978 (0.947-1.010)	0.171		
Male	0.883 (0.321-2.432)	0.810		
Secondary prophylaxis	1.925 (0.774-4.790)	0.159		
Ischemic etiology	0.469 (0.178-1.235)	0.125		
Previously diagnosed atrial fibrillation/atrial flutter	1.291 (0.507-3.285)	0.592		
Hypertension	0.709 (0.268-1.871)	0.487		
Diabetes mellitus	0.681 (0.225-2.054)	0.495		
Stroke/TIA	0.045 (0.000-154.999)	0.455		
Bradypacing indication	0.588 (0.136-2.548)	0.478		
LVEF	0.979 (0.943-1.017)	0.283		
Heart rate	0.995 (0.969-1.022)	0.726		
Creatinine	1.001 (0.993-1.010)	0.798		
eGFR	1.013 (0.988-1.038)	0.308		
Hemoglobin	1.005 (0.980-1.031)	0.699		
Antiplatelets	0.851 (0.345-2.096)	0.726		
Anticoagulation	1.036 (0.421-2.552)	0.938		
Beta-blocker	0.483 (0.064-3.653)	0.481		
RAAS	1.740 (0.232-13.059)	0.590		
Diuretics	0.634 (0.255-1.576)	0.326		
MRA	0.556 (0.223-1.385)	0.208		
Digitalis	1.024 (0.137-7.673)	0.982		
CCB	0.570 (0.076-4.275)	0.585		
Amiodaron	0.151 (0.020-1.133)	**0.066**	0.151 (0.020-1.133)	0.066
Statins	0.592 (0.241-1.459)	0.255		
SGLT2-inhibitors	0.041 (0.000-56.696)	0.387		

HR: hazard ratio, CI: confidence interval, TIA: transient ischemic attack, LVEF: left ventricular ejection fraction, eGFR: estimated glomerular filtration rate, RAAS: renin-angiotensin-aldosterone system, MRA: mineralocorticoid receptor antagonist, CCB: calcium channel blocker, SGLT2-inhibitor: sodium-glucose cotransporter-2.

Journal of
Clinical Medicine

MDPI

Table S4. Multivariate analysis of time to first inappropriate therapy – dual-chamber discriminator VDD vs. dual-chamber discriminator DDD.

Risk factor	unadjusted HR (95% CI)	p-value	adjusted HR (95% CI)	p-value
ICD type (VDD or DDD)	0.586 (0.230-1.490)	0.262	0.597 (0.226-1.579)	0.299
Age at implantation	0.976 (0.945-1.008)	0.133		
Male	0.834 (0.300-2.319)	0.729		
Secondary prophylaxis	1.918 (0.757-4.862)	0.170		
Ischemic etiology	0.389 (0.139-1.092)	**0.073**	0.311 (0.101-0.953)	**0.041**
Previously diagnosed atrial fibrillation/atrial flutter	1.187 (0.444-3.169)	0.733		
Hypertension	0.710 (0.266-1.898)	0.495		
Diabetes mellitus	0.528 (0.153-1.828)	0.314		
Stroke/TIA	0.045 (0.000-310.033)	0.493		
Bradypacing indication	0.581 (0.133-2.531)	0.470		
LVEF	0.977 (0.938-1.018)	0.264		
Heart rate	0.998 (0.972-1.024)	0.863		
Creatinine	1.001 (0.991-1.011)	0.826		
eGFR	1.017 (0.991-1.044)	0.200		
Hemoglobin	1.006 (0.980-1.033)	0.643		
Antiplatelets	0.895 (0.355-2.257)	0.814		
Anticoagulation	0.935 (0.371-2.358)	0.888		
Beta-blocker	0.457 (0.060-3.470)	0.449		
RAAS	1.480 (0.197-11.140)	0.704		
Diuretics	0.537 (0.212-1.360)	0.190		
MRA	0.458 (0.181-1.161)	**0.100**	0.682 (0.252-1.846)	0.452
Digitalis	1.091 (0.145-8.200)	0.932		
CCB	0.664 (0.088-4.989)	0.690		
Amiodaron	0.031 (0.000-2.684)	0.127		
Statins	0.525 (0.208-1.324)	0.172		
SGLT2-inhibitors	0.042 (0.000-97.771)	0.422		

HR: hazard ratio, CI: confidence interval, TIA: transient ischemic attack, LVEF: left ventricular ejection fraction, eGFR: estimated glomerular filtration rate, RAAS: renin-angiotensin-aldosterone system, MRA: mineralocorticoid receptor antagonist, CCB: calcium channel blocker, SGLT2-inhibitor: sodium-glucose cotransporter-2.

Journal of
Clinical Medicine

Table S5. Multivariate analysis of time to first appropriate therapy – single-chamber vs. dual-chamber.

Risk factor	unadjusted HR (95% CI)	p-value	adjusted HR (95% CI)	p-value
Discrimination algorythm	0.724 (0.428-1.224)	0.228	0.699 (0.389-1.257)	0.232
Age at implantation	1.008 (0.989-1.027)	0.421		
Male	1.605 (0.865-2.975)	0.133		
Secondary prophylaxis	2.810 (1.744-4.527)	**<0.001**	2.641 (1.560-4.471)	**<0.001**
Ischemic etiology	1.976 (1.223-3.191)	**0.005**	2.214 (1.309-3.745)	**0.003**
Previously diagnosed atrial fibrillation/atrial flutter	0.955 (0.592-1.541)	0.850		
Hypertension	1.627 (0.875-3.026)	0.124		
Diabetes mellitus	1.333 (0.823-2.158)	0.243		
Stroke/TIA	1.602 (0.734-3.496)	0.236		
Bradypacing indication	0.409 (0.177-0.942)	**0.036**	0.348 (0.126-0.960)	**0.042**
LVEF	0.999 (0.983-1.016)	0.935		
Heart rate	0.992 (0.979-1.006)	0.277		
Creatinine	1.002 (0.997-1.006)	0.485		
eGFR	0.993 (0.981-1.005)	0.259		
Hemoglobin	0.989 (0.976-1.002)	**0.091**	0.998 (0.984-1.011)	0.738
Antiplatelets	1.627 (1.025-2.582)	**0.039**	1.260 (0.667-2.382)	0.476
Anticoagulation	0.941 (0.598-1.481)	0.793		
Beta-blocker	2.489 (0.346-17.924)	0.365		
RAAS	1.536 (0.620-3.808)	0.354		
Diuretics	0.848 (0.529-1.360)	0.495		
MRA	0.941 (0.576-1.538)	0.809		
Digitalis	0.420 (0.103-1.710)	0.226		
CCB	1.016 (0.466-2.215)	0.968		
Amiodaron	2.065 (1.301-3.279)	**0.002**	1.751 (1.053-2.911)	**0.031**
Statins	1.520 (0.911-2.538)	0.109		
SGLT2-inhibitors	0.341 (0.107-1.088)	**0.069**	0.358 (0.086-1.492)	0.158

HR: hazard ratio, CI: confidence interval, TIA: transient ischemic attack, LVEF: left ventricular ejection fraction, eGFR: estimated glomerular filtration rate, RAAS: renin-angiotensin-aldosterone system, MRA: mineralocorticoid receptor antagonist, CCB: calcium channel blocker, SGLT2-inhibitor: sodium-glucose cotransporter-2.

Journal of
Clinical Medicine

MDPI

Table S6. Multivariate analysis of all-cause mortality – single-chamber vs. dual-chamber.

Risk factor	unadjusted HR (95% CI)	p-value	adjusted HR (95% CI)	p-value
Discrimination algorythm	0.930 (0.598-1.448)	0.749	0.714 (0.426-1.197)	0.201
Age at implantation	1.034 (1.019-1.050)	**<0.001**	1.027 (1.009-1.045)	**0.003**
Male	2.315 (1.373-3.903)	**0.002**	2.290 (1.326-3.956)	**0.003**
Secondary prophylaxis	0.677 (0.473-0.970)	**0.034**	0.826 (0.541-1.262)	0.376
Ischemic etiology	1.723 (1.218-2.438)	**0.002**	1.339 (0.923-1.942)	0.124
Previously diagnosed atrial fibrillation/atrial flutter	1.151 (0.807-1.641)	0.438		
Hypertension	1.833 (1.149-2.926)	**0.011**	1.432 (0.864-2.372)	0.164
Diabetes mellitus	1.654 (1.164-2.350)	**0.005**	1.223 (0.820-1.824)	0.323
Stroke/TIA	1.817 (1.023-3.229)	**0.042**	1.356 (0.714-2.575)	0.353
Bradypacing indication	1.006 (0.636-1.593)	0.979		
LVEF	0.966 (0.951-0.982)	**<0.001**	0.969 (0.951-0.986)	**<0.001**
Heart rate	1.001 (0.991-1.011)	0.822		
Creatinine	1.004 (1.001-1.006)	**0.004**	1.000 (0.993-1.007)	0.985
eGFR	0.986 (0.977-0.994)	**<0.001**	1.000 (0.989-1.011)	0.975
Hemoglobin	0.982 (0.972-0.991)	**<0.001**	0.982 (0.972-0.992)	**<0.001**
Antiplatelets	1.339 (0.952-1.883)	**0.093**	0.987 (0.604-1.613)	0.959
Anticoagulation	1.196 (0.850-1.682)	0.304		
Beta-blocker	0.439 (0.214-0.900)	**0.025**	0.250 (0.120-0.525)	**<0.001**
RAAS	1.656 (0.810-3.387)	0.167		
Diuretics	2.468 (1.576-3.867)	**<0.001**	1.209 (0.697-2.098)	0.500
MRA	1.765 (1.156-2.695)	**0.008**	0.796 (0.460-1.375)	0.413
Digitalis	1.447 (0.759-2.756)	0.262		
CCB	0.380 (0.155-0.929)	**0.034**	0.192 (0.047-0.785)	**0.022**
Amiodaron	1.438 (0.999-2.072)	**0.051**	1.239 (0.834-1.842)	0.288
Statins	1.498 (1.029-2.181)	**0.035**	1.110 (0.703-1.754)	0.654
SGLT2-inhibitors	1.159 (0.580-2.316)	0.676		

HR: hazard ratio, CI: confidence interval, TIA: transient ischemic attack, LVEF: left ventricular ejection fraction, eGFR: estimated glomerular filtration rate, RAAS: renin-angiotensin-aldosterone system, MRA: mineralocorticoid receptor antagonist, CCB: calcium channel blocker, SGLT2-inhibitor: sodium-glucose cotransporter-2.

Journal of
Clinical Medicine

MDPI

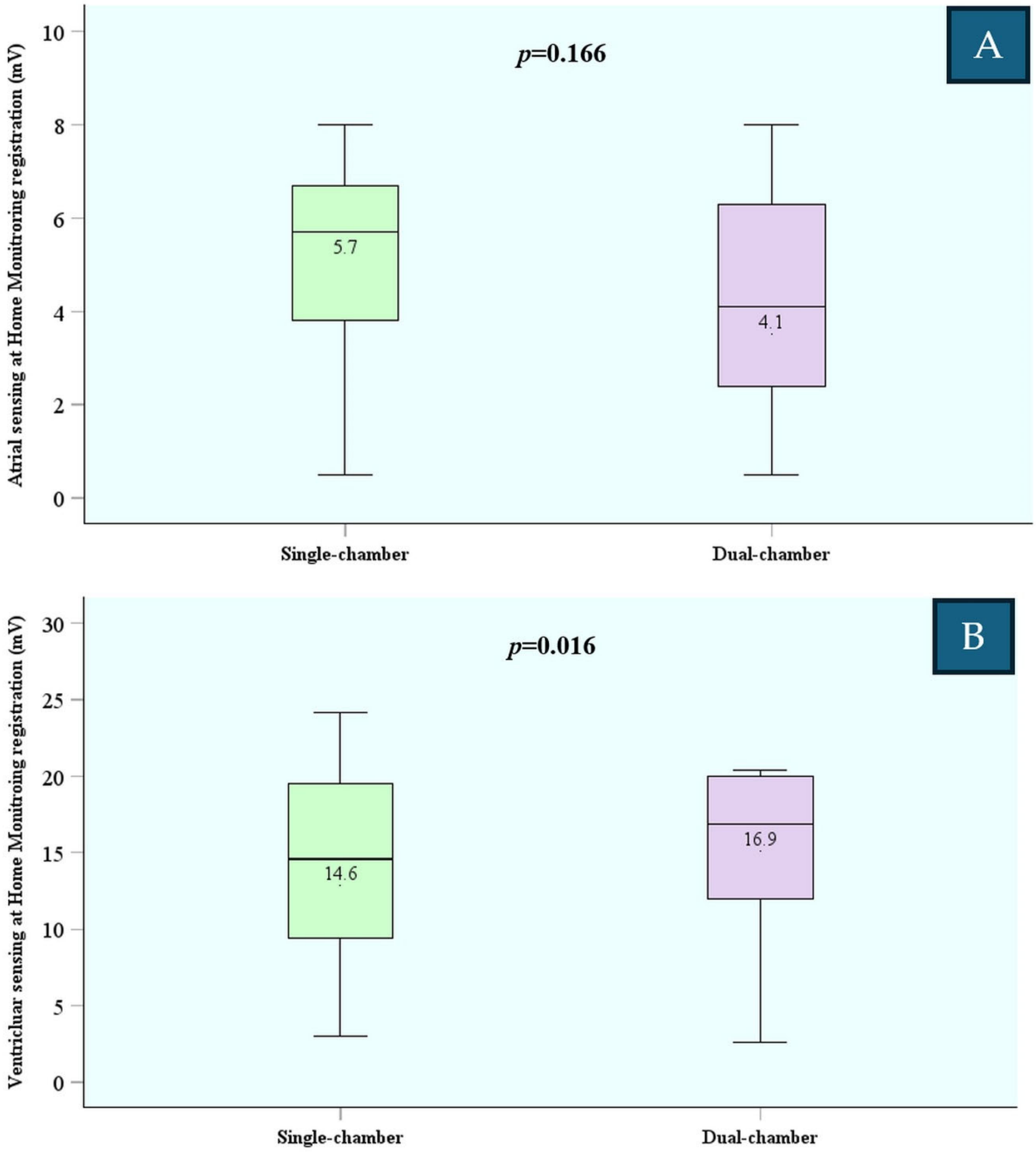

Figure S1. (A) Baseline atrial sensing parameters in single-chamber and dual-chamber discrimination groups. **(B)** Baseline ventricular sensing parameters in single-chamber and dual-chamber discrimination groups.

Journal of Clinical Medicine

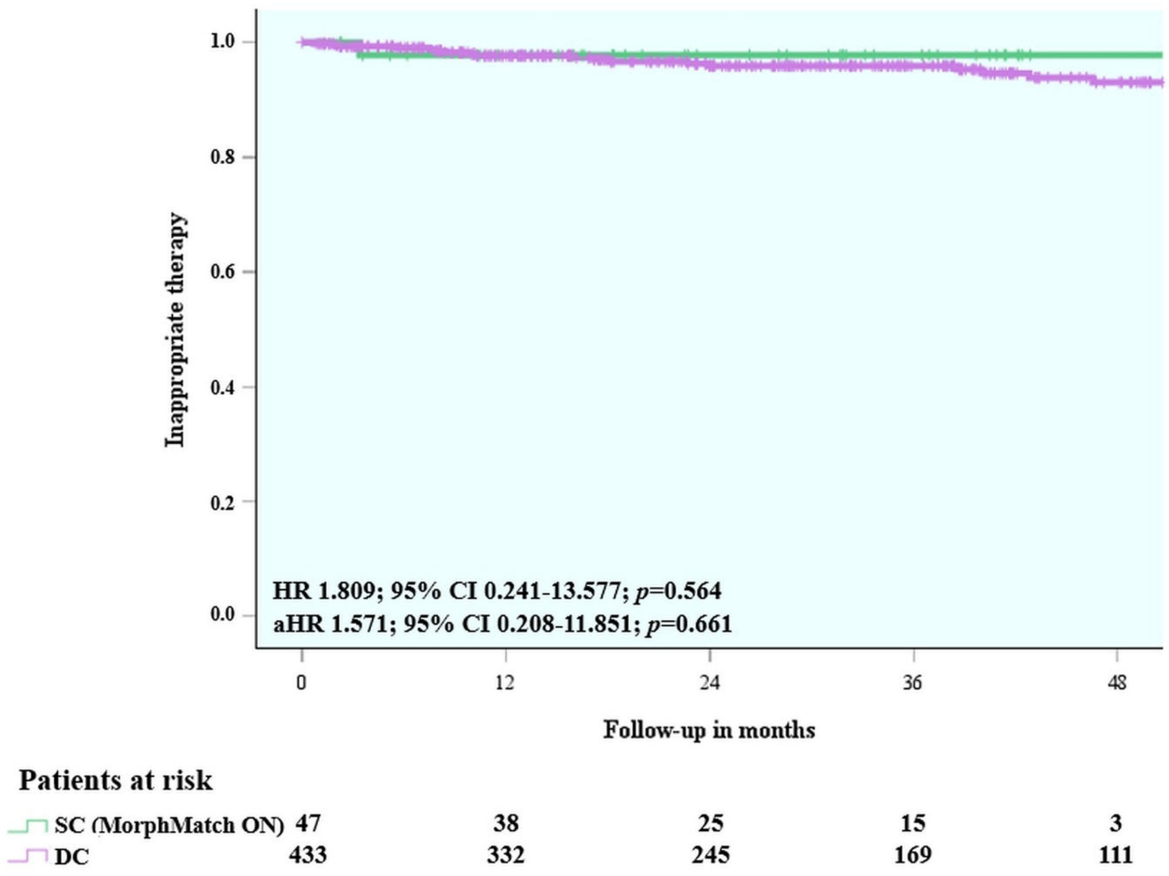

Figure S2. Time to first inappropriate therapy – single-chamber (MorphMatch ON) vs. dual-chamber.

HR: hazard ratio, aHR: adjusted hazard ratio, CI: confidence interval, SC: single-chamber, DC: dual-chamber.

Journal of
Clinical Medicine

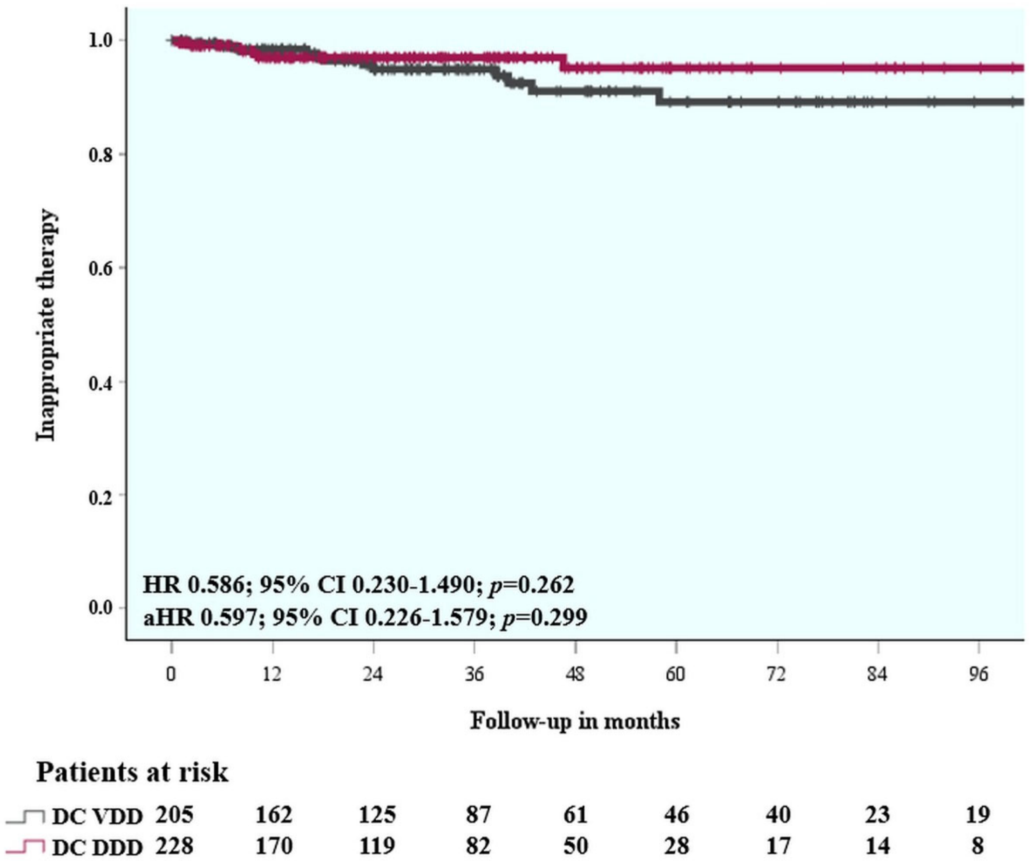

Figure S3. Time to first inappropriate therapy – dual-chamber discriminator VDD vs. dual-chamber discriminator DDD.

HR: hazard ratio, aHR: adjusted hazard ratio, CI: confidence interval, DC: dual-chamber, VDD: single-lead ICD device with a floating atrial dipole, DDD: dual-chamber ICD.

Journal of
Clinical Medicine

MDPI

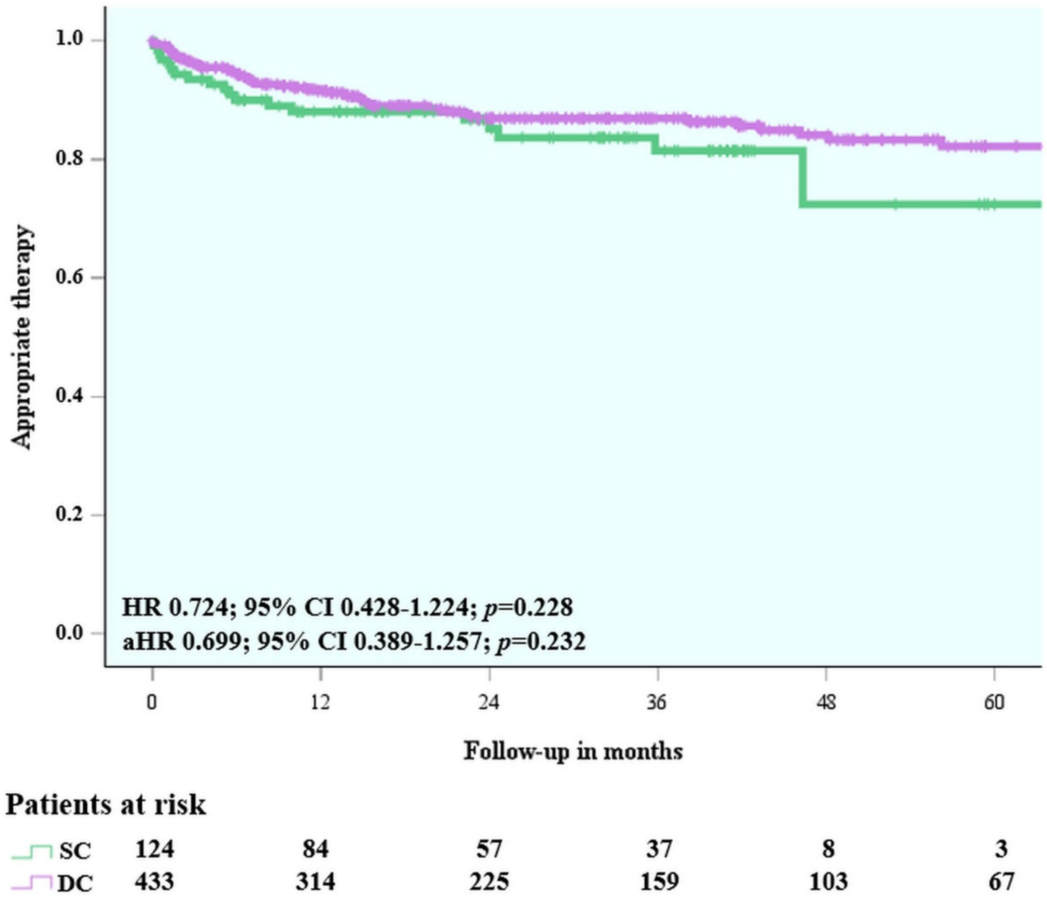

Figure S4. Time to first appropriate therapy – single-chamber vs. dual-chamber. HR: hazard ratio, aHR: adjusted hazard ratio, CI: confidence interval, SC: single-chamber, DC: dual-chamber.

Journal of
Clinical Medicine

MDPI

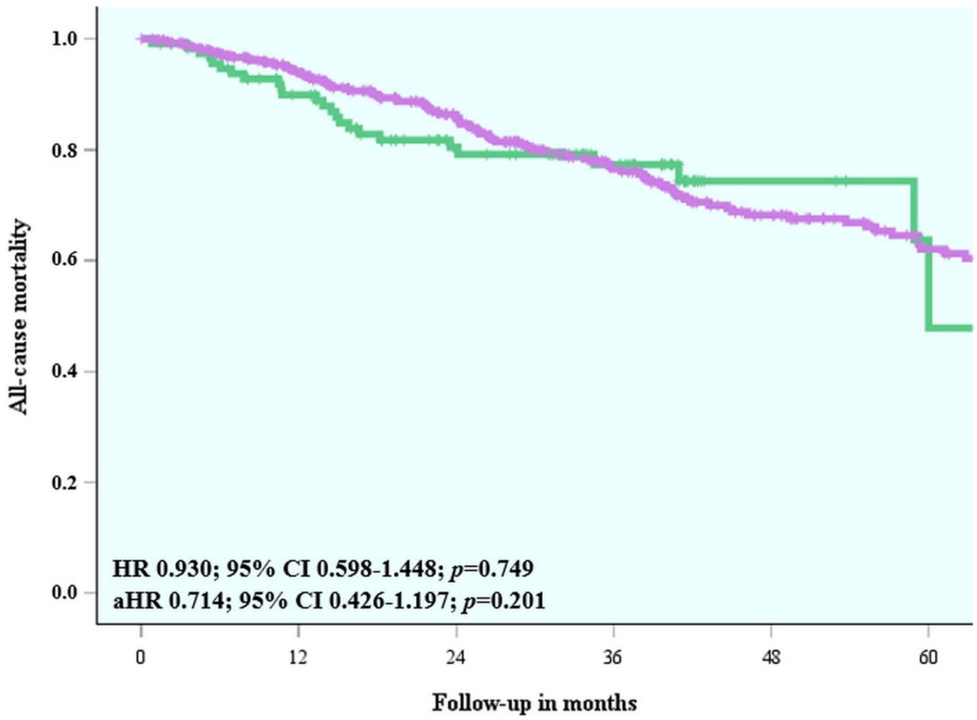

Patients at risk

SC	116	92	62	42	9	4
DC	414	328	247	171	111	75

Figure S5. All-cause mortality – single-chamber vs. dual-chamber.
HR: hazard ratio, aHR: adjusted hazard ratio, CI: confidence interval, SC: single-chamber, DC: dual-chamber.
